# Supplementary material for: Learning curve and surgical outcome of robotic assisted colorectal surgery with ERAS program
Source: Sci Rep. 2022 Nov 29;12:20566. doi: 10.1038/s41598-022-24665-w (PMC9709162; doi:10.1038/s41598-022-24665-w)
Supplement: Supplementary file 1 — Supplementary Information 1. [file 41598_2022_24665_MOESM1_ESM.zip › LSC Raw data-Ñ[▒K/148/N_ñw╝╨░O▒Kñσ.pdf]

W52 / W52

臺中榮民總醫院
手術室護理記錄

[redacted]

基本資料

- 室別： 二醫開刀房-31室，手術科別： CRS，手術類別： 常規，日期： 2022/01/06
- 進入時間： 1625 ； 麻醉開始時間： 1625 ； Time Out時間： 1710 ； 劃刀時間： 1710 ； 手術結束時間： 2210 送出時間： 2215 ；
- 麻醉方式： GE

  診斷：CECAL CA WITH HIGH CEA RO LUNG METS

  手術名稱：Laparoscopic right colectomy and anastomosis

  手術局部用藥：nil

  術前預防性抗生素：
  - 抗生素名稱一： Veterin 1GM IVD/由麻醉護理執行
  - 時間： 1655
  - 上次簽章: 彭馨慧

  ，追加預防性抗生素：
- 手術醫師： 蔣鋒帆，楊曙榮，林志安
- 麻醉醫師： VS 徐煥
- 刷手護士A ： 許晏妮 ，時間： 1625 ，接替刷手一： 陳昱穎 ，時間： 1730 ，接替刷手二： 洪紹瓊 ，時間： 2100

  流動護士A ： 彭馨慧 ，時間： 1625 ，接替流動一： 許晏妮 ，時間： 1800 ，接替流動二： 謝昀安 ，時間： 2100

皮膚狀況

A-V Shunt： 無

術前： 不完整
- 7. 破皮

部位(術前)：

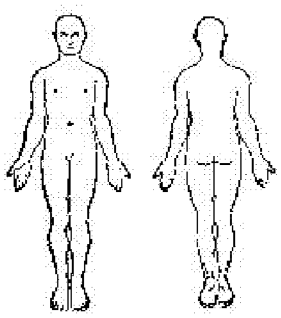

左大腿內側破皮

記錄者： 上次簽章: 彭馨慧

術後： 不完整
- 7. 破皮

部位(術後)：

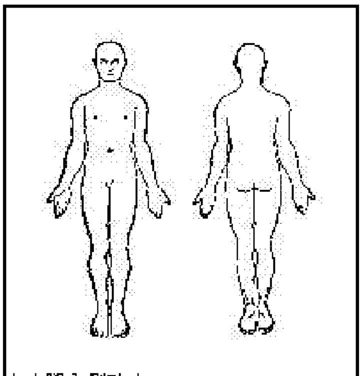

記錄者： 謝昀安 上次簽章: 許晏妮

手術準備

手術臥式 單一手術臥式

-
    - 手術臥式： 仰臥
    - 使用附件： 溫毯 38 ℃，頭圈
    - 皮膚準備： 10%Aq Betadine，Hibiscrub，2% Chlorhexidine
    - 電燒： 使用電燒: 電極片位置： 右大腿，記錄者： 上次簽章: 彭馨慧

止血帶T： 無

植入物

- surgiwrap/in pelvic/壹/0.02*100*130mm/LOT54779
  Ethicon endo-surgery/on colon/75mm/NTLC75

, 記錄者: 謝昀安 上次簽章: 許晏妮

標本送檢

- 細菌培養 無
- 細胞學 無
- 冰凍切片 無
- 病理標本
  組織名稱一：A colon
- 截肢 無

標本交付 標本： 壹 袋，標本： 零 盒，標本： 零 瓶，細胞學： 零 管，Culture Tube： 零 管，被交付醫師： R 林志安，

- 記錄者： 謝昀安 上次簽章: 許晏妮
- 時間： 2200

傷口術後敷料

傷口敷料：

- 請於圖上標示部位：

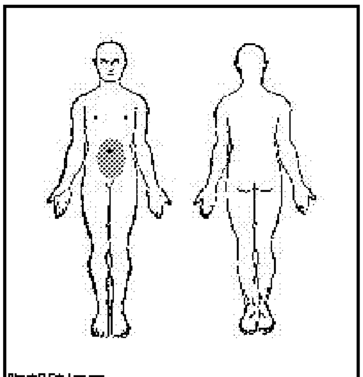

- 傷口敷料： MEPORE

導管

導管	天數	導管資訊	插入日期	插入時間	更換日期	拔除日期	拔除時間	拔除原因	預計拔除/更換	預計拔除/更換日期
A-line (1)	1	號碼:20 號; 部位:左撓動脈;	2022/01/06	1710						
Double-J(1)	1	號碼:6FR.x26CM ; 部位:右側輸尿管;	2022/01/06	2030						
Foley(1)	1	號碼:16 Fr.; 部位:膀胱; 註記:水球10CC;	2022/01/06	1700						

用物計數

- 手術中出血量： MINIMAL c.c.
- 手術中尿量： 400 c.c.
-
  - 其它： 無

用物計數： 多切口同時縫合

- 1.器械： 正確，2.縫針： 正確，3.刀片： 正確，4.紗布： 正確，5.腹部墊： 未使用，6.紗球： 未使用，7.PEANUTS ： 未使用，8.紗條： 未使用，9.絲帶： 未使用，10.針頭： 未使用，11.Vessel loop： 未使用，12.Boots： 未使用，13.Jelco： 未使用，14.棉條： 未使用，
  - 第一次：
    - 刷手護士 ： 陳昱穎
    - 流動護士： 上次簽章: 許晏妮
    - 時間： 2000

  ,
  - 第二次：
    - 刷手護士 ： 陳昱穎
    - 流動護士： 上次簽章: 許晏妮
    - 時間： 2020

  ,
  - 第三次：
    - 刷手護士 ： 洪紹瓊
    - 流動護士： 謝昀安 上次簽章: 許晏妮
    - 時間： 2110

備註

備註：NIL

- 記錄者： 謝昀安 上次簽章: 許晏妮
- 時間： 2215

填寫護理人員 謝昀安

正式摘要

W52 / W52

doctorRank:B
